# A Sum-of-Squares and Semidefinite Programming Approach for Maximum Likelihood DOA Estimation

**DOI:** 10.3390/s16122191

**Published:** 2016-12-20

**Authors:** Shu Cai, Quan Zhou, Hongbo Zhu

**Affiliations:** 1 Jiangsu Key Laboratory of Wireless Communication, Nanjing University of Posts and Telecommunications, Nanjing 210003, China; zhuhb@njupt.edu.cn; 2 Key Laboratory of Ministry of Education for Broad Band Communication and Sensor Network Technology, Nanjing University of Posts and Telecommunications, Nanjing 210003, China; quan.zhou@njupt.edu.cn; 3 School of Computer and Software, Nanjing University of Information Science and Technology, Nanjing 210044, China

**Keywords:** DOA estimation, maximum likelihood, uniform linear array, sum-of-squares, semidefinite programming, alternating projection

## Abstract

Direction of arrival (DOA) estimation using a uniform linear array (ULA) is a classical problem in array signal processing. In this paper, we focus on DOA estimation based on the maximum likelihood (ML) criterion, transform the estimation problem into a novel formulation, named as *sum-of-squares* (SOS), and then solve it using semidefinite programming (SDP). We first derive the SOS and SDP method for DOA estimation in the scenario of a single source and then extend it under the framework of alternating projection for multiple DOA estimation. The simulations demonstrate that the SOS- and SDP-based algorithms can provide stable and accurate DOA estimation when the number of snapshots is small and the signal-to-noise ratio (SNR) is low. Moveover, it has a higher spatial resolution compared to existing methods based on the ML criterion.

## 1. Introduction

Estimating the direction of arrivals (DOAs) of multiple plane waves using passive arrays is one of the central problems in radar, sonar, radio astronomy, and wireless communication. In the last two decades, many methods have been proposed for solving this problem [1,2]. In existing literature, DOA estimation based on maximum likelihood (ML) criterion can achieve the optimal estimation performance [1,2], but requires solving a multidimensional optimization problem, which is nonlinear, non-convex, and computationally intensive. The alternating projection (AP) technique [3] is proposed to replace the multidimensional optimization problem by a sequence of one-dimensional optimization subproblems, which are still nonlinear and non-convex. To reduce the computational complexity, the subspace-based methods, such as multiple signal classification (MUSIC) [4], estimation of signal parameters via rotational invariance technique (ESPRIT) [5], and RootMUSIC [2], are proposed. These methods are efficient and can approach the optimal performance asymptotically. However, theoretical analysis and simulation results show that subspace methods usually exhibit a certain performance loss in estimating locations of highly correlated signals [6]. Results in [7,8] demonstrate that subspace methods may also suffer from performance loss in active localization scenarios. Using the structure of uniform linear array (ULA) array manifold, the authors of [9,10] have proposed an iterative quadratic maximum likelihood (IQML) method to solve the ML problem. An improvement over IQML is introduced in [6,11]. The resulting algorithm is called method of direction estimation (MODE) and achieves the asymptotic accuracy of the true optimum with a closed form solution [2].

Recently, DOA estimation methods based on compressed sensing have been proposed in [12,13,14,15,16,17,18,19,20]. The compressed sensing-based methods enjoy a lot of virtues. For example, they are robust to coherent signals, can estimate DOAs with only one snapshot, and detect the number of unknown signals automatically. In general, compressed sensing methods can be divided into three categories: on-grid model-based methods [12,17], grid-based off-grid methods [13,16,18], and gridless methods [14,15,19,20]. On-grid model-based methods choose a fixed discrete grid in the continuous domain of directions as the set of DOA estimates, and assume that the true DOAs are exactly on the grid. It is obvious that the on-grid assumption cannot be satisfied in practice and thus on-grid methods are approximation methods. Grid-based off-grid methods parameterize the errors between true (off-grid) DOAs and grid points and then estimate them together with on-grid variables. They are more robust to errors induced by off-grid DOAs. However, these methods are still grid-based methods, whose performance depends on the trade-off between the grid size and the computational workload [15]. Gridless sparse methods avoid the off-grid problem completely. Moreover, they are guaranteed to generate a sparse estimation with a high probability under moderate conditions. However, they may suffer from spurious estimates and lower spatial resolution [15,20]. The reweighted atomic norm minimization (RAM) approach can alleviate these problems when the signal-to-noise ratio (SNR) is large [19].

In this work, we will focus on the ML criterion. First, we consider estimating the DOA of a single source and show that the corresponding ML problem can be formulated into a univariate polynomial optimization problem, which can further be transformed into an semidefinite programming (SDP) [21] and solved efficiently by using interior point methods (IPM) [22]. Then, the proposed algorithm is extended to the scenario of multiple DOA estimation under the framework of AP. Compared with the existing methods, the proposed method can provide more stable and accurate DOA estimates when the SNR is low and/or the number of snapshots is small. Moreover, it achieves a higher spatial resolution.

The rest of the paper is organized as follows. The array signal model and the ML estimation problem are formulated in Section 2. Section 3 introduces the proposed method in detail. Performance of the proposed method is demonstrated by simulations in Section 4. Then, Section 5 concludes the paper.

Notation: in the paper, the superscripts *T* and *H* denote the transpose and transpose conjugate, respectively. The superscripts (r) and (i) are used to denote the real and imaginary part of a complex parameter, respectively. $S_{+}^{N}$ denotes the set of *N*-dimensional symmetric positive semidefinite matrices and $R^{M\times N}$ the set of M×N real matrices.

## 2. Modeling and Problem Statement

### 2.1. Array Signal Model

Consider an *N*-element ULA which is illuminated by *M*(<*N*) far-field narrow band sources. The received array signal vector is denoted by:

(1) x(t)=A(θ)s(t)+n(t)

where $s\left(t\right)={\left[s_{1}\left(t\right),\ldots ,s_{M}\left(t\right)\right]}^{T}$ is the signal vector, n(t) denotes the additive noise vector, $A\left(\theta \right)=\left[a\left(\theta_{1}\right),\ldots ,a\left(\theta_{M}\right)\right]$ is the array manifold, and $a(\theta_{m})$ is the steering vector of the *m*-th source. For a ULA, the steering vector $a(\theta_{m})$ of source *m* has the following form:

(2) $$a\left(\theta_{m}\right)={\left[1,e^{j\frac{2\pi }{\lambda }d\;\sin\left(\theta_{m}\right)},\ldots ,e^{j\frac{2\pi }{\lambda }\left(N-1\right)d\;\sin\left(\theta_{m}\right)}\right]}^{T}$$

where *d* and *λ* denote the element spacing and the wavelength of the working frequency, respectively.

The following assumptions are required for the subsequent ML estimation of unknown parameters:

1. The noise n(t) is stationary and Gaussian distributed with zero mean, $E\left[n\left(t\right)n^{H}\left(t\right)\right]=\sigma_{n}^{2}I$, and $E\left[n\left(t\right)n^{T}\left(t\right)\right]=0$, where I is the identity matrix.
2. The signals are uncorrelated with the noise.

### 2.2. Maximum Likelihood Parameter Estimation

According to above-mentioned assumptions, the log-likelihood function of Equation (1) is given by:

(3) $$\begin{array}{cc} L & =-MN_{t}\ln\sigma_{n}^{2}-\frac{1}{\sigma_{n}^{2}}\sum_{k=1}^{N_{t}}∥x\left(t_{k}\right)-A\left(\theta \right)s\left(t_{k}\right){∥}^{2} \end{array}$$

where $N_{t}$ is the number of snapshots. Maximizing Equation (3) with respect to $\sigma_{n}^{2}$ leads to the following optimization problem [23]:

(4) $$\begin{array}{cc} {\left\{\hat{\theta },\hat{S}\right\}}_{\mathrm{ML}} & =\arg\;\;\min_{\theta ,S}\sum_{k=1}^{N_{t}}∥x\left(t_{k}\right)-A\left(\theta \right)s\left(t_{k}\right){∥}^{2}\\ & =\arg\;\;\min_{\theta ,S}∥X-A\left(\theta \right)S{∥}^{2} \end{array}$$

where $X=[x\left(t_{1}\right),\ldots ,x\left(t_{N_{t}}\right)]$ and $S=[s\left(t_{1}\right),\ldots ,s\left(t_{N_{t}}\right)]$. The ML estimate of S can be obtained straightforwardly from Equation (4), given ***θ***, and is denoted by $\hat{S}={\left(A^{H}\left(\theta \right)A\left(\theta \right)\right)}^{-1}A^{H}\left(\theta \right)X$. Substituting this estimate back into Equation (4), we have the ML DOA estimation performed by:

(5) $$\begin{array}{c} \hat{\theta }=\arg\;\;\max_{\theta }\;\;\mathrm{tr}\left\{{\hat{R}}_{x}P_{A(\theta )}\right\} \end{array}$$

where ${\hat{R}}_{x}=\frac{1}{N_{t}}XX^{H}$ and $P_{A(\theta )}=A\left(\theta \right){\left(A^{H}\left(\theta \right)A\left(\theta \right)\right)}^{-1}A^{H}\left(\theta \right)$ is the projection matrix in the column space of A(θ).

## 3. The SOS and SDP Based DOA Estimation Approach

In this part, we will firstly derive the sum-of-squares (SOS) and SDP approach to solve problem Equation (5) with M=1 and then extend the method to the M≥1 case under the framework of alternating projection (AP).

### 3.1. Estimate the DOA of a Single Signal Source

When M=1, the ML DOA estimation problem Equation (5) is reduced to

(6) $$\begin{array}{c} \hat{\theta }=\arg\;\;\max_{\theta }a^{H}\left(\theta \right){\hat{R}}_{x}a\left(\theta \right) \end{array}$$

We first transform problem Equation (6) to a univariate polynomial fractional function optimization problem by the following variable replacement. Define $v=\frac{\pi }{\lambda }d\sin\left(\theta \right)$ and t=tan(v). Then, the (k+1)-th element of a(θ) can be written as:

(7) $$\begin{array}{c} e^{j\frac{2\pi }{\lambda }kd\sin\left(\theta \right)}={\left(e^{j2v}\right)}^{k}={\left[\cos\left(2v\right)+j\sin\left(2v\right)\right]}^{k} \end{array}$$

Substituting the trigonometric identities $\cos\left(2v\right)=\frac{1-t^{2}}{1+t^{2}}$ and $\sin\left(2v\right)=\frac{2t}{1+t^{2}}$ into Equation (7) yields a fractional polynomial with variable *t*:

(8) $$\begin{array}{c} e^{j\frac{2\pi }{\lambda }kd\sin\left(\theta \right)}=\frac{{\left(1-t^{2}+2jt\right)}^{k}}{{\left(1+t^{2}\right)}^{k}}=\frac{h_{k}^{\left(r\right)}\left(t\right)+jh_{k}^{\left(i\right)}\left(t\right)}{{\left(1+t^{2}\right)}^{k}} \end{array}$$

where $h_{k}^{\left(r\right)}\left(t\right)$ and $h_{k}^{\left(i\right)}\left(t\right)$ denote the real and imaginary parts of ${\left(1-t^{2}+2jt\right)}^{k}$, respectively. For ease of expression, let us assume $d=\frac{\lambda }{2}$, such that the bijection $\theta =\arcsin(\frac{2}{\pi }\arctan\left(t\right))$ is monotonic when t∈R and *θ* varies within $\left[-\frac{\pi }{2},\frac{\pi }{2}\right]$. Notice that when $d>\frac{\lambda }{2}$, the range of *θ* is decreased to $\left[-\arcsin\left(\frac{\lambda }{2d}\right),\arcsin\left(\frac{\lambda }{2d}\right)\right]$ for t∈R.

Denote the (i,j)th entry of ${\hat{R}}_{x}$ as $r_{i,j}$, substitute Equation (8) into a(θ) and then a(θ) into Equation (6). The objective function of Equation (6) can be equivalently transformed as follows:

(9) aH(θ)R^xa(θ)=∑i=1Nri,i+2Re∑k=2N∑i=kNr(i−k+1),iej(k−1)2v=m1+2Re∑k=2Nmkej(k−1)2v=m1+2∑k=2Nmk(r)hk−1(r)(t)−mk(i)hk−1(i)(t)(1+t2)k−1

where $m_{k}=m_{k}^{\left(r\right)}+jm_{k}^{\left(i\right)}=\sum_{i=k}^{N}r_{(i-k+1),i}$ and “Re{·}” denotes the real part of a complex number. By omitting the constants in Equation (9), the problem Equation (6) is equivalent to the following optimization problem:

(10) $$\begin{array}{c} \max_{t\in R}\sum_{k=2}^{N}\frac{m_{k}^{\left(r\right)}h_{k-1}^{\left(r\right)}\left(t\right)-m_{k}^{\left(i\right)}h_{k-1}^{\left(i\right)}\left(t\right)}{{\left(1+t^{2}\right)}^{k-1}} \end{array}$$

By defining the following polynomials:

(11) $$\begin{array}{cc} f_{1}\left(t\right) & :={\left(1+t^{2}\right)}^{N-1}=\sum_{i=1}^{2N-1}a_{i}t^{2N-1-i} \end{array}$$

(12) $$\begin{array}{cc} f_{2}\left(t\right) & :=\sum_{k=2}^{N}\left[\left(m_{k}^{\left(r\right)}h_{k-1}^{\left(r\right)}\left(t\right)-m_{k}^{\left(i\right)}h_{k-1}^{\left(i\right)}\left(t\right)\right){\left(1+t^{2}\right)}^{N-k}\right]\\ & =\sum_{i=1}^{2N-1}b_{i}t^{2N-1-i} \end{array}$$

The problem Equation (10) can be briefly expressed as:

(13) $$\begin{array}{c} \max_{t\in R}\;f_{2}\left(t\right)/f_{1}\left(t\right) \end{array}$$

which is a univariate polynomial fractional function optimization problem.

Then, we will solve problem Equation (13) by two steps: Finding its optimal objective function value and then solving the optimal solution. Note that the first step can be performed by solving the following problem:

(14) $$\begin{array}{cc} \min_{p} & \;\;p\\ s.t. & \;\;p\geq f_{2}\left(t\right)/f_{1}\left(t\right),\;\forall t\in R \end{array}$$

Since $f_{1}\left(t\right)>0,\forall t\in R$, Equation (14) is equivalent to:

(15) $$\begin{array}{cc} \min_{p} & \;\;p\\ s.t. & \;\;pf_{1}\left(t\right)-f_{2}\left(t\right)\geq 0,\;\forall t\in R \end{array}$$

It is well known that a univariate polynomial is nonnegative over the real domain if and only if it can be written as an SOS (see [24] and references therein). This means that the constraint in problem Equation (15) is equivalent to $\exists Z\in S_{+}^{N}$, such that [21]:

(16) $$\begin{array}{c} t^{T}Zt=pf_{1}\left(t\right)-f_{2}\left(t\right)=\sum_{i=1}^{2N-1}\left(pa_{i}-b_{i}\right)t^{2N-1-i},\;\;\forall t\in R \end{array}$$

where $t=[1,t,\cdots ,t^{N-1}]$ is a Vandermonde vector and the second equation is based on Equations (11) and (12). Since the coefficients of $t^{k}$ and ∀k on both sides of Equation (16) are equal, the identical Equation (16) contains a bunch of equality constraints. With these constraints, problem Equation (15) can be equivalently written as the following semidefinite programming (SDP):

(P1) $$\begin{array}{cc} \min_{p,Z} & \;\;p\\ s.t. & \;\;pa_{2N-1-k}-b_{2N-1-k}=\mathrm{tr}\left\{ZH^{\left(N,k+1\right)}\right\}\\ & \;\;k=0,1,\cdots ,2N-2\\ & \;\;Z⪰0 \end{array}$$

where “Z⪰0” indicates that Z is a positive semidefinite matrix and $H^{\left(N,k\right)}\in R^{N\times N}$ is a Hankel matrix with the (i,j)th entry

(17) $$\begin{array}{c} H_{i,j}^{\left(N,k\right)}=\left\{\begin{array}{cc} 1, & \mathrm{if}\;i+j=k+1\\ 0, & \mathrm{otherwise} \end{array}\right. \end{array}$$

Problem (P1) can be solved by using IPM like SDPT3 [25].

In the second step, we find the optimal solution of Equation (13). Denote the optimal solution of (P1) as $p^{*}$ and $Z^{*}$. Then, the optimal *t* must satisfy $p^{*}f_{1}\left(t\right)-f_{2}\left(t\right)=0$, or, equivalently, $t^{T}Z^{*}t=0$. This is equivalent to finding a *t* such that the Vandermonde vector t is in the null space of $Z^{*}$, i.e., solving the equation

(18) $$\begin{array}{c} Z^{*}t=0 \end{array}$$

Denote the null space of $Z^{*}$ as $N(Z^{*})$, its rank as $r_{n}$, and $t^{*}={\left[1,t^{*},\cdots ,t^{*(N-1)}\right]}^{T}$ as the solution of Equation (18). If $r_{n}=1$, $t^{*}$ is a scaled version of the unique base vector of $N(Z^{*})$, which can be denoted as $z_{n}$, and $t^{*}$ can be obtained by $t^{*}=z_{n}\left(2\right)/z_{n}\left(1\right)$. If $r_{n}>1$, the rank of $Z^{*}$ is $N-r_{n}$, which means that Equation (18) contains $N-r_{n}$ independent equations. Using Gaussian elimination to these equations, we can finally obtain an equation with the order of $r_{n}$. Solve this equation and choose the root which maximizes $f_{2}\left(t\right)/f_{1}\left(t\right)$ as $t^{*}$. With $t^{*}$, the ML estimate of DOA can be obtained by:

(19) $$\begin{array}{c} \hat{\theta }=\arcsin\left(2\arctan\left(t^{*}\right)/\pi \right) \end{array}$$

We refer to the above ML-based single DOA estimation method as sum-of-squares and semidefinite programming approach (SOS-SDP). This algorithm can provide a global optimal solution for the DOA estimation problem Equation (6) with a worst case complexity of $O(N^{6.5})$ [26]. However, it may be numerically unstable. For example, assume the number of antennas is N=20 and t=0.1. The algorithm requires an accuracy of at least $10^{-19}$ to express $t^{19}$. Therefore, SOS-SDP will be inaccurate or fail as the number of antenna elements becomes large. We here propose two compensation strategies to improve its robustness and accuracy.

1. When $r_{n}=1$, calculate $t_{i}^{*}=z_{n}\left(i+1\right)/z_{n}\left(i\right)$ for i=1,⋯,N−1; when $r_{n}>1$, perform Gaussian elimination procedure $N-r_{0}$ times such that the obtained equations keep the *i*-th to $\left(i+r_{0}\right)$-th order of *t* for $i=1,\cdots ,N-r_{0}$, respectively, and calculate the roots of all the $\left(N-r_{0}\right)$ equations. Then, choose the $t_{i}^{*}$ or root that maximizes $\frac{f_{2}\left(t\right)}{f_{1}\left(t\right)}$ as $t_{0}^{*}$ and obtain the corresponding $\theta_{0}^{*}$ by Equation (19).
2. Using $\theta_{0}^{*}$ as an initial point, minimize $f\left(\theta \right)=-\mathrm{tr}\left\{P_{A(\theta )}{\hat{R}}_{x}\right\}$ by using the Newton’s iteration in Algorithm 1.

**Algorithm 1** The Procedure of One-Dimensional Newton’s Iteration.**Input:**  A small positive constant, $\epsilon_{0}$; the maximum number of iterations, *K*; an initial point, $\theta_{0}^{*}$; **Output:**  An estimate of DOA, $\theta^{*}$; 1:  k=0 and $\theta_{0}=\theta_{0}^{*}$; 2:  **repeat** 3:      $d_{k}=-{\left(f^{\prime \prime }\left(\theta_{k}\right)\right)}^{-1}f^{\prime }\left(\theta_{k}\right)$; 4:      Choose a step size $\alpha_{k}$ using *Armijo* rule [27]; 5:      $\theta_{k+1}=\theta_{k}+\alpha_{k}d_{k}$; 6:      $\epsilon =f^{\prime }\left(\theta_{k}\right)d_{k}$ and k=k+1; 7:  **until**
$\epsilon <\epsilon_{0}$ or k>K; 8:  **return**
$\theta^{*}=\theta_{k}$.

For the one-dimensional search problem above with an initial point very close to the optimal value, the Newton’s iteration converges in several iterations and the cost of each iteration is very small.

### 3.2. Estimate DOAs of Multiple Signal Sources

In this section, we extend SOS-SDP proposed in Section 3.1 to estimate multiple DOAs in the framework of AP [3]. Note that SOS-SDP applies to a single DOA estimation and cannot estimate multiple DOAs simultaneously. On the other hand, AP transforms a multiple DOA estimation problem into a sequence of one DOA estimation subproblems.

For ease of expression, we have listed the procedure of AP in Algorithm 2, where:

(20) $$\begin{array}{cc} {\hat{\theta }}_{m}^{\left(0\right)} & =[{\hat{\theta }}_{1}^{\left(0\right)},\cdots ,{\hat{\theta }}_{m-1}^{\left(0\right)}]\\ {\hat{\theta }}_{m}^{\left(k\right)} & =[{\hat{\theta }}_{1}^{\left(k\right)},\cdots ,{\hat{\theta }}_{m-1}^{\left(k\right)},{\hat{\theta }}_{m+1}^{\left(k-1\right)},\cdots ,{\hat{\theta }}_{M}^{\left(k-1\right)}]\\ A_{m}^{\left(k\right)} & =\left[A\left({\hat{\theta }}_{m}^{\left(k\right)}\right),a\left(\theta_{m}\right)\right] \end{array}$$

where ${\hat{\theta }}_{m}^{\left(k\right)}$ denotes the estimate of $\theta_{m}$ at the *k*th iteration, k=1,⋯,K, and *K* is the specified maximum number of iterations.

**Algorithm 2** The Framework of Alternating Projection Based on ML Criterion.**Input:**  A small positive constant, *ϵ*; the maximum number of iterations, *K*; **Output:**  DOA estimates, $\theta_{m}^{*}$, m=1,2,⋯,M; 1:  k=0; 2:  **for**
m=1; m≤M; m++
**do** 3:  ${\hat{\theta }}_{m}^{\left(k\right)}=\arg\max_{\theta_{m}}\mathrm{tr}\left\{{\hat{R}}_{x}P_{A_{m}^{\left(k\right)}}\right\}$; 4:  **end for** 5:  **repeat** 6:  k=k+1; 7:  **for**
m=1; m≤M; m++
**do** 8:    ${\hat{\theta }}_{m}^{\left(k\right)}=\arg\max_{\theta_{m}}\mathrm{tr}\left\{{\hat{R}}_{x}P_{A_{m}^{\left(k\right)}}\right\}$; 9:  **end for** 10:  **until**
k>K or $\sum_{m=1}^{M}\left|{\hat{\theta }}_{m}^{\left(k\right)}-{\hat{\theta }}_{m}^{\left(k-1\right)}\right|<\epsilon ;$ 11:  **return**
$\theta_{m}^{*}={\hat{\theta }}_{m}^{\left(k\right)}$, m=1,2,⋯,M.

According to step 1 in Algorithm 2, SOS-SDP proposed in Section 3.1 can be used directly when k=0 and m=1. For the cases k+m>1, we need to solve the problem listed in both step 1 and step 2 of Algorithm 2:

(21) $$\begin{array}{c} {\hat{\theta }}_{m}^{\left(k\right)}=\arg\max_{\theta_{m}}\;\mathrm{tr}\left\{{\hat{R}}_{x}P_{A_{m}^{\left(k\right)}}\right\} \end{array}$$

where $P_{A_{m}^{\left(k\right)}}=A_{m}^{\left(k\right)}{\left({\left(A_{m}^{\left(k\right)}\right)}^{H}A_{m}^{\left(k\right)}\right)}^{-1}{\left(A_{m}^{\left(k\right)}\right)}^{H}$. Substituting Equation [3]:

$$P_{A_{m}^{\left(k\right)}}=P_{A({\hat{\theta }}_{m}^{\left(k\right)})}+P_{a{\left(\theta_{m}\right)}_{A({\hat{\theta }}_{m}^{\left(k\right)})}}$$

into Equation (21) yields the following problem:

(22) $$\begin{array}{c} {\hat{\theta }}_{m}^{\left(k\right)}=\arg\max_{\theta_{m}}\frac{a^{H}\left(\theta_{m}\right)P_{A({\hat{\theta }}_{m}^{\left(k\right)})}^{⊥}{\hat{R}}_{x}P_{A({\hat{\theta }}_{m}^{\left(k\right)})}^{⊥}a\left(\theta_{m}\right)}{a^{H}\left(\theta_{m}\right)P_{A({\hat{\theta }}_{m}^{\left(k\right)})}^{⊥}a\left(\theta_{m}\right)} \end{array}$$

where $a{\left(\theta \right)}_{A({\hat{\theta }}_{m}^{\left(k\right)})}=P_{A({\hat{\theta }}_{m}^{\left(k\right)})}^{⊥}a\left(\theta \right)$ and $P_{A({\hat{\theta }}_{m}^{\left(k\right)})}^{⊥}=I-P_{A({\hat{\theta }}_{m}^{\left(k\right)})}$.

Note that Equation (22) has a structure similar to Equation (6). Therefore, inserting variable replacement Equations (8)–(22), and, following the same steps as Equations (9)–(12), one can easily cast the optimization problem Equation (22) into a univariate polynomial optimization problem, which has the same structure as Equation (13). Then, SOS-SDP proposed in Section 3.1 is applicable.

### 3.3. Complexity Analysis

The main costs of SOS-SDP come from two parts: estimating the data covariance matrix, whose complexity is $O(N^{2}N_{t})$, and solving the SDP problem (P1) in each iteration, whose worst case complexity is $O(N^{6.5})$, as mentioned in Section 3.1. Therefore, the worst case complexity of SOS-SDP under the framework of AP is $O(N^{2}N_{t}+KMN^{6.5})$.

## 4. Results

In this section, we demonstrate the performance of SOS-SDP by comparing it with some existing methods, which include RootMUSIC [1], MODE [6], IQML [10], sparse and parametric approach (SPA) [15], greedy block coordinate descent algorithm (GBCD) [17], weighted GBCD (GBCD+) [17], atomic norm minimization (ANM) [20] , reweighted atomic-norm minimization (RAM) [19], and AP based on exhaustive search (AP) [3]. Note that RootMUSIC is a subspace-based method, MODE, IQML, and SOS-SDP are based on the ML criterion, and SPA, GBCD, ANM, and RAM are sparse methods. The Matlab (2013b script, The MathWorks Inc., Natick, MA, USA) codes of SPA and ANM are both available online [28]. RootMUSIC and IQML are based on the Matlab built-in functions “rootmusic” and “phased.RootWSFEstimator” with default settings. The Cramer–Rao bound (CRB) for the DOA estimation is also given as a benchmark (see (8.102) in [1]). The parameters of SOS-SDP in Algorithm 2 are chosen as $\epsilon =10^{-4}$ and the maximum number of iterations is K=10. Note that K=10 is large enough for AP [3]. The parameters for Newton’s iteration in Algorithm 1 are chosen as $\epsilon_{0}=10^{-10}$ with a maximum number of iterations of 10. The signal model is described in Equations (1) and (2), where the distance between array elements is chosen as d=λ/2. Complex white Gaussian noise is added to the array output with noise variance $\sigma_{n}^{2}$. The SNR (in dB) is defined as $10\log_{10}\left(p_{1}/\sigma_{n}^{2}\right)$, where $p_{1}=E(∥s_{1}\left(t\right){∥}^{2})$ and E[·] is the expectation of a variable.

**Figure 1:**
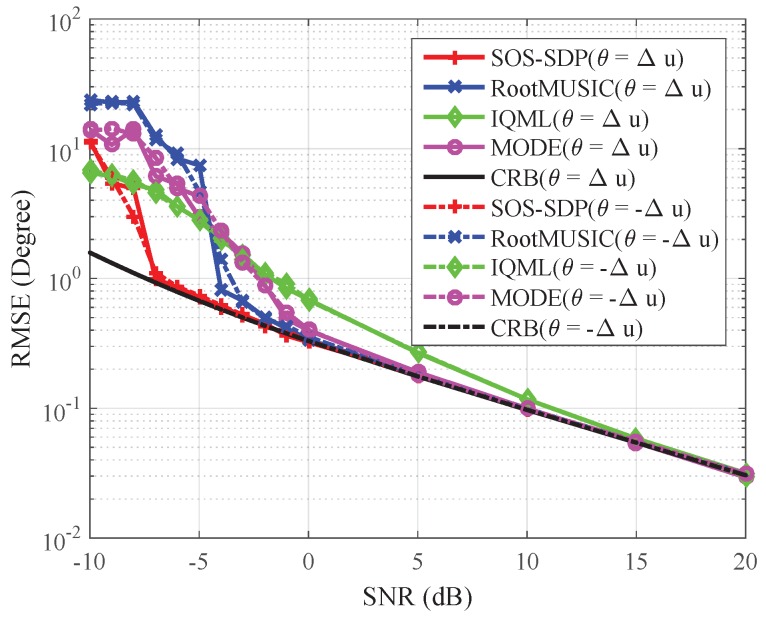
Comparison of RMSE of different methods and the CRB. Some settings include: ULA with N=12 and M=2 equal power uncorrelated sources with θ=[Δu,−Δu], and the number of snapshots $N_{t}=100$.

The first experiment considers estimating DOAs of uncorrelated signal sources. Two equal-power independent signal sources located at $\theta_{1}=\Delta u$ and $\theta_{2}=-\Delta u$ are impinging on a standard 12-element ULA, where $\Delta u=\frac{0.2165}{2}BW_{\mathrm{NN}}$ and $BW_{\mathrm{NN}}=2\arcsin\left(\frac{2}{N}\right)$ radians denote bandwidth between the first nulls in the spatial spectrum [1]. The number of snapshots is $N_{t}=100$. The root mean square errors (RMSEs) of the methods based on the subspace and ML criteria are calculated. The results are shown in Figure 1, where T=1000 Monte Carlo simulations are performed at each SNR, the RMSE of $\theta_{i}$ is calculated by

(23) $$RMSE_{i}=\frac{1}{T}\sum_{t=1}^{T}{\left(\theta_{i}-{\hat{\theta }}_{i,t}\right)}^{2},\;\;i=1,2$$

and ${\hat{\theta }}_{i,t}$ is the estimate of $\theta_{i}$ in the *t*-th simulation. In the figure, one can easily see that the RMSEs of all the methods are decreased with the increase of SNR. When SNR ≥−7 dB, the RMSE of SOS-SDP coincides with the CRB. However, with the decreasing of SNR, this RMSE increases sharply. This behavior is referred to as the threshold phenomenon [1]. The threshold of the SOS-SDP algorithm is about 3 dB, 6 dB, and more than 10 dB lower than those of RootMUSIC, MODE, and IQML, respectively. According to this result, SOS-SDP can provide a better estimation accuracy at the low SNR region. The reason for this superiority might be SOS-SDP, which solves the ML problem Equation (5) directly, providing a global optimal solution not only for DOAs, but also for signals and noise variance (which are estimated implicitly). This means that all of the unknown variables in the ML problem are jointly and optimally tuned. However, MODE and RootMUSIC estimate noise variance (or noise subspace) firstly and then find optimal DOAs based on these estimates. Therefore, their DOA estimates depend on the noise variance (or noise subspace) estimations, which might be inaccurate when the SNR or the number of snapshots is limited. DOA estimates obtained via IQML are almost always inconsistent and have larger mean squared errors [10].

In the second experiment, we consider estimating DOAs of two coherent signal sources, where the correlation coefficient between the two sources is ρ=1. Since the subspace-based method does not work in this case, we only compare the methods based on the ML criterion. Two different scenarios are considered: the first one sets the number of snapshots as $N_{t}=100$ and varies the SNR, while the second one lets SNR = 0 dB and changes the number of snapshots. The rest parameters are similar to those of the first experiment.

**Figure 2:**
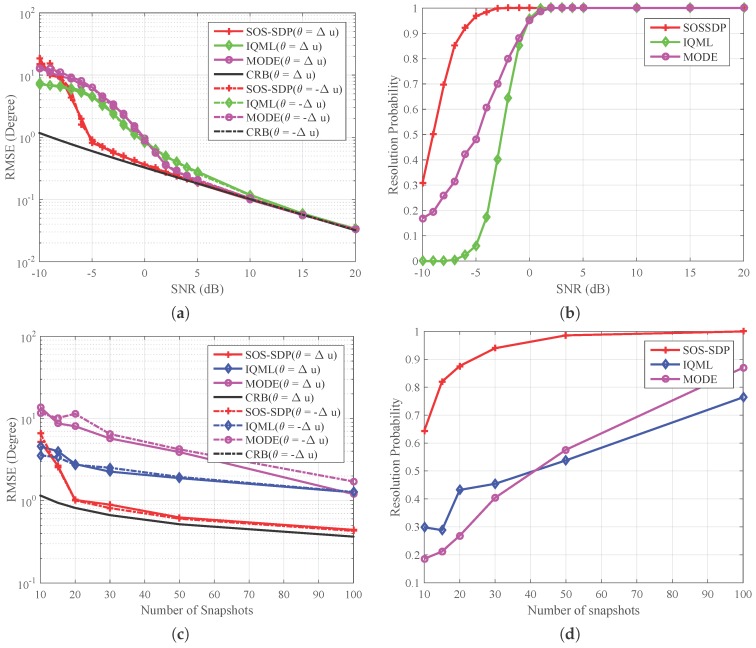
Comparisons of RMSE and resolution probability of different methods for two equal power coherent sources. Other settings include: θ=[Δu,−Δu] and ULA with N=12. (**a**) RMSE versus SNR with $N_{t}=100$; (**b**) resolution probability versus SNR with $N_{t}=100$; (**c**) RMSE versus the number of snapshots with SNR =0 dB; and (**d**) resolution probability versus the number of snapshots with SNR =0 dB.

Figure 2a illustrates the RMSE performance of three different methods against SNR. We can see that, for SNR ≥−5 dB, the RMSE of SOS-SDP approaches the CRB. When SNR <−5 dB, the threshold phenomenon occurs and the RMSE increases rapidly. This threshold is lower than those of MODE and IQML, which are SNR =2 dB and SNR =5 dB, respectively. The corresponding resolution probabilities against SNR are given in Figure 2b. The two sources are said to be resolvable [29,30] if $|{\hat{\theta }}_{i}-\theta_{i}\left|\leq \right|\theta_{1}-\theta_{2}|/2$ for both i=1,2, where ${\hat{\theta }}_{i}$ denotes the estimate of $\theta_{i}$. We can see that the resolution probabilities of different methods are enhanced with the increase of SNR. The resolution probability of the proposed method approaches 1 for SNR larger than −5 dB, while those of the rest of the methods approach 1 at 0 dB. This result coincides with the RMSE performance in Figure 2a.

Figure 2c illustrates the RMSE performance of three different methods varying with the number of snapshots. We can see that although the SNR is small, the proposed method can achieve a good estimation performance and approach the CRB with a small number of snapshots. Conversely, the remaining two cannot provide an effective DOA estimation even when the number of snapshot becomes larger. The corresponding resolution probability is shown in Figure 2d. It is seen that the resolution probability of SOS-SDP approaches 1 when the number of snapshots is larger than 30, while the rest of them are smaller than 0.9 with 100 snapshots. According to the trends of the curves in Figure 2c,d, one can expect that MODE can achieve good estimation performance when the number of snapshot is large. This coincides with the conclusion that MODE is a large sample realization of the ML method [6]. Based on the results shown in the four figures, we can conclude that the proposed method can provide a stable and accurate DOA estimation with a small number of snapshots and at low SNR level. The reason for this virtue may be that subproblems in each iteration are solved optimally and thus the ML problem is solved optimally.

In the third experiment, spatial resolution of the proposed method is tested. To achieve this, we change the distance between signal sources and compare detection performance of SOS-SDP with those of SPA and the methods based on the ML criterion. Consider two equal power signal sources impinging on a 10-element ULA. The signals are coherent with each other and the correlation coefficient is ρ=exp(−jπ/4). They are located at $\theta_{1}=\Delta \theta /2$ and $\theta_{1}=-\Delta \theta /2$, respectively, with Δθ varying from 0.02 $BW_{\mathrm{NN}}$ to 0.2 $BW_{\mathrm{NN}}$. The number of snapshots and the SNR are set as 100 and 10 dB, respectively.

**Figure 3:**
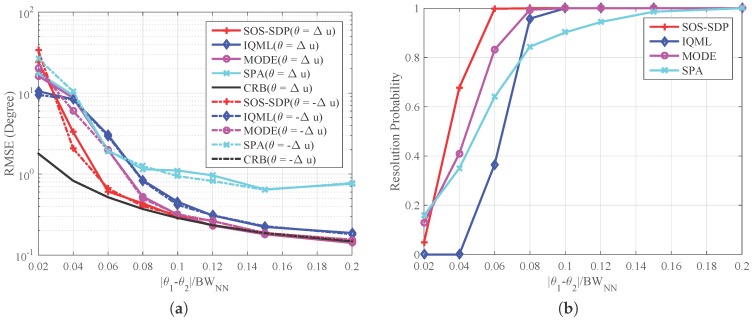
Spatial resolution of different methods with two equal-power coherent sources. Other settings include: ULA with N=10, number of snapshots $N_{t}=100$, and SNR =10 dB (**a**) RMSE versus distance between sources; and (**b**) resolution probabilities versus distance between sources.

Figure 3a shows RMSE curves against Δθ. It is seen that the RMSEs of different methods are decreased with the increasing of Δθ. The corresponding resolution probabilities are shown in Figure 3b, which approach 1 as Δθ becomes larger. Based on the results of the two figures, we can see that SOS-SDP can distinguish two signal sources with probability of 1 at Δθ≥ 0.06 $BW_{\mathrm{NN}}$, where its RMSE also approaches CRB. This bound of Δθ for MODE and IQML are 0.08 $BW_{\mathrm{NN}}$ and 0.1 $BW_{\mathrm{NN}}$, respectively. It is also seen that the RMSE of SPA cannot approach the CRB even when its resolution probability approaches 1. The reason may be that SPA does not use the knowledge of *M* [15]. Hence, SOS-SDP has the highest spatial resolution according to the simulation results. The average running time of SOS-SDP, IQML, MODE and SPA are 15.03 s, 0.18 s, 0.12 s, and 1.1 s. The complexity of the proposed method is higher than that of others, which is a cost for the better estimation performance. We should mention that IQML and MODE are built-in functions of Matlab, while the rest are based on CVX (Version 2.0) [31], whose execution efficiency can be further improved.

The fourth experiment compares SOS-SDP with AP based on exhaustive search and some sparse methods developed recently, i.e., GBCD [17], weighted GBCD (GBCD+) [17], ANM [20], and RAM [19]. Note that GBCD and GBCD+ are on-grid model-based methods and ANM and RAM are gridless methods. GBCD and GBCD+ are implemented as in [17] except that the gird size is 2000 and the maximum number of iterations equals the number of antennas. RAM is implemented as in [19] except that the number of signals is given. This is because RAM may underestimate the number of signals when SNR is small. The grid size for exhaustive search in each step of AP is 10,000. To save computational time, we initialize SOS-SDP by AP with grid size 1000. Three independent signal sources are located at $\theta_{1}=-\frac{180\Delta u}{\pi }+0.1$, $\theta_{2}=\frac{180\Delta u}{\pi }+0.1$, and $\theta_{3}=\frac{180BW_{\mathrm{NN}}}{\pi }$ degrees, respectively. Note that AP may provide an RMSE lower than CRB if $\theta_{1}=-\theta_{2}$. The numbers of antennas and snapshots are 12 and 200, respectively.

**Figure 4:**
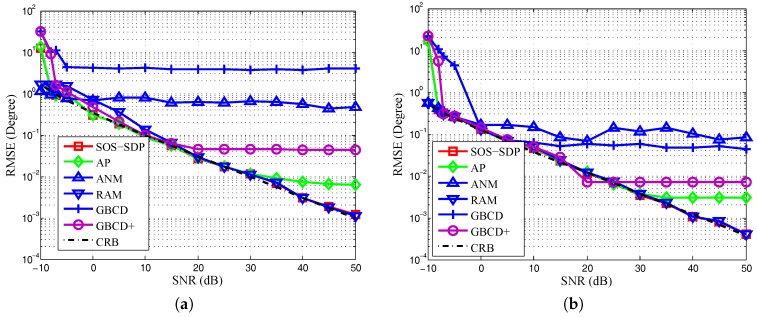
Comparison of RMSE of different methods and the CRB with N=12, $N_{t}=100$, and M=3 equal power uncorrelated sources. (**a**) RMSE of estimation of $\theta_{1}$ versus SNR; (**b**) RMSE of estimation of $\theta_{3}$ versus SNR.

Figure 4a,b illustrates the RMSEs of DOA estimations of $\theta_{1}$ and $\theta_{3}$, respectively. The RMSE of $\theta_{2}$ is similar to that of $\theta_{1}$ and omitted here. In the figures, we can see that when SNR is small, AP performs similarly to SOS-SDP, and with the increasing of SNR, the RMSEs of grid-based methods, i.e., AP and GBCD+, are lower bounded by some constants, respectively. These constants depend on the size of the grid. The weighted sparse methods (GBCD+ and RAM) outperform their unweighted versions (GBCD and ANM) in RMSE, respectively. RAM is the best in the compared sparse methods. However, in Figure 4a, RAM still cannot approach CRB when two signals are closely spaced and SNR is not large. This is because the estimates of RAM tend to merge together when SNR is small [19]. The average running times of AP, SOS-SDP (initialized by AP), GBCD (and GBCD+), ANM, and RAM are about 0.3 s, 2.5 s, 3.5 s, 3 s, and 6 s, respectively. AP with exhaustive search performs similarly to SOS-SDP with less time when SNR is small. However, it is a grid-based method, whose performance is based on the trade-off between grid size and computational workload. As a result, SOS-SDP might be faster than AP if a dense grid is adopted in exhaustive search for obtaining high accuracy. Moreover, the complexity order of SOS-SDP might be decreased if there are more sophisticated algorithms.

## 5. Conclusions

We propose an SOS formulation for the ML DOA estimation problem with ULAs and solve it by using an SDP approach. The proposed method can provide a stable and accurate DOA estimation with a small number of snapshots and low SNR. Moreover, it has a higher spatial resolution than the existing methods. The proposed method is slow compared to the existing ML-based methods, since the cost of solving the SDP in each iteration is extremely high. A future work is to develop faster solvers for the SDPs involved in this paper. Since the alternating direction method of multipliers (ADMM) [32] may provide an acceptable solution with a smaller cost, we may turn to the first-order method in future studies. Another interesting direction will be extending the proposed DOA estimation method to other kinds of arrays, such as uniform circular arrays.
